# Determining the True Polarity and Amplitude of Synaptic Currents Underlying Gamma Oscillations of Local Field Potentials

**DOI:** 10.1371/journal.pone.0075499

**Published:** 2013-09-20

**Authors:** Gonzalo Martín-Vázquez, Julia Makarova, Valeri A. Makarov, Oscar Herreras

**Affiliations:** 1 Department of Systems Neuroscience, Cajal Institute – Consejo Superior de Investigaciones Científicas, Madrid, Spain; 2 Department of Applied Mathematics, Faculty of Mathematics, Universidad Complutense de Madrid, Madrid, Spain; University College of London - Institute of Neurology, United Kingdom

## Abstract

Fluctuations in successive waves of oscillatory local field potentials (LFPs) reflect the ongoing processing of neuron populations. However, their amplitude, polarity and synaptic origin are uncertain due to the blending of electric fields produced by multiple converging inputs, and the lack of a baseline in standard AC-coupled recordings. Consequently, the estimation of underlying currents by laminar analysis yields spurious sequences of inward and outward currents. We devised a combined analytical/experimental approach that is suitable to study laminated structures. The approach was essayed on an experimental oscillatory LFP as the Schaffer-CA1 gamma input in anesthetized rats, and it was verified by parallel processing of model LFPs obtained through a realistic CA1 aggregate of compartmental units. This approach requires laminar LFP recordings and the isolation of the oscillatory input from other converging pathways, which was achieved through an independent component analysis. It also allows the spatial and temporal components of pathway-specific LFPs to be separated. While reconstructed Schaffer-specific LFPs still show spurious inward/outward current sequences, these were clearly stratified into distinct subcellular domains. These spatial bands guided the localized delivery of neurotransmitter blockers in experiments. As expected, only Glutamate but not GABA blockers abolished Schaffer LFPs when applied to the active but not passive subcellular domains of pyramidal cells. The known chemical nature of the oscillatory LFP allowed an empirical offset of the temporal component of Schaffer LFPs, such that following reconstruction they yield only sinks or sources at the appropriate sites. In terms of number and polarity, some waves increased and others decreased proportional to the concomitant inputs in native multisynaptic LFPs. Interestingly, the processing also retrieved the initiation time for each wave, which can be used to discriminate afferent from postsynaptic cells in standard spike-phase correlations. The applicability of this approach to other pathways and structures is discussed.

## Introduction

Local field potentials (LFPs) are raised by population synaptic currents and typically display irregular behavior interspersed with epochs of prominent oscillatory activity that are concentrated in narrow frequency bands [1]. Computationally, LFP-oscillations can be viewed as temporal windows to precisely control the timing of converging pathways. They may also have a role in the formation of neuron assemblies [2]. Notably, significant fluctuations in the amplitude, duration and spatial localization of successive LFP-waves are observed that reflect the rich internal dynamics of the afferent and target populations [3,4,5,6,7]. In the monolayered hippocampus, the bulk of currents is generated by a single target population [7,8,9,10], but there may be more in the cortex [11]. Reading amplitude fluctuations in LFP-waves requires an understanding of the number and nature of the synaptic pathway/s from which they originate (i.e., single or multiple, excitatory or inhibitory). Classical ambiguities regarding the localization and synaptic nature of the current sources underlying LFPs impede a straightforward interpretation of these fluctuations [12]. Also, phase relationships between LFP-wave and spike trains, which are widely used in the literature to establish cause-effect relationships rarely allow one to determine whether the firing unit is pre- or postsynaptic to LFPs.

Although the theoretical bases of LFP generation are well established [13,14,15,16,17,18,19], this topic is rarely explored directly due to the significant difficulties in resolving the inverse problem of identifying the neuronal current sources from LFPs with subcellular precision. Indeed, the number of co-activated afferent populations at a given instant is unknown [7]. Moreover, most modern amplifiers reject the DC component of LFPs and as a result, defining the polarity of the AC-coupled LFP-oscillations is precluded by the lack of a baseline, which in turn frustrates the determination of the excitatory or inhibitory nature of the underlying synaptic currents. As a consequence, one cannot set a time reference for the initiation of each LFP-cycle, which is necessary to establish the phase of the ongoing fluctuations.

**Figure 1:**
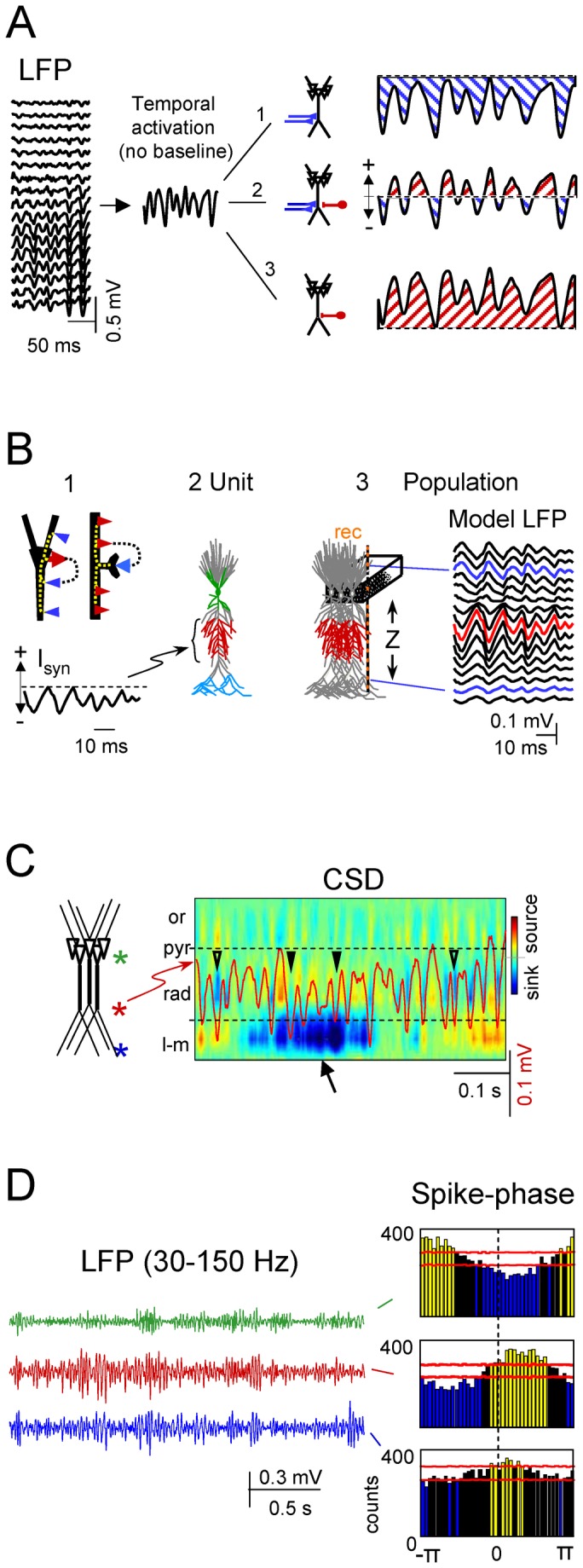
Illustration of the problem and the limitations for the reading of fluctuations in oscillatory LFPs. Linear LFPs recorded in standard AC-coupled mode along the vertical axis of the CA1 hippocampus (A). The epoch displays rhythmic gamma waves with different amplitudes. The oscillatory nature and the removal of slow-components by AC-coupling eliminate the true baseline and impede determining the excitatory (1), inhibitory (3), or dual (2) nature of the underlying currents. B: Single cell-to-aggregate scaling of synaptic currents (forward solution for model LFPs)(1). Whether inhibitory (red arrow in the soma) or excitatory (blue arrow in spine), the synaptic currents set transmembrane loops that contain both inward and outward currents distributed throughout the cell. 2-3 show the scaling of single cell currents to population LFPs using a realistic multineuronal CA1 model. For testing purposes, we used anatomically separated inputs (color coded). In the example, the pyramidal cell (2) was injected with a rhythmic sequence of excitatory synaptic currents (I_syn_) at a dendritic domain, and the estimated currents in all compartments were fed into the aggregate model (3) from which the LFPs were calculated along the main cell axis (Z). The complex morphology of individual cells led to strong asymmetrical distribution of macroscopic LFPs (arrows). C shows an LFP trace recorded at the st. *radiatum* (in red) superimposed to the spatiotemporal map of CSD. Note the variable and poor matching of currents and gamma waves (arrowheads) in the synaptic Schaffer domain (between the dashed lines), caused by a variable offset due to the strong currents in other domains (arrow). *Or*, stratum oriens; *pyr*, pyramidal; *rad*., *radiatum*; *l-m*, lacunosum-molecular. D shows different spike-phase histograms for a single spike train from a unit recorded in the CA1 and LFPs simultaneously recorded at three different sites (marked by asterisks in **c**). Horizontal red lines mark the confidence limits: only the yellow and blue bars are statistically significant.

In laminated brain structures with stratified inputs, such as the cortex and hippocampus, the polarity of underlying transmembrane currents can theoretically be estimated from the spatial gradients of the extracellular field potential [13] through current source-density (CSD) analysis [20,21]. CSD maps are free of volume-conducted currents from remote cell generators. Therefore, this analysis identifies membrane domains that produce a net flow of inward or outward currents (sinks and sources, respectively), which can then be matched to anatomical data to determine whether a given domain is associated with synaptic sites or with passive counterparts. While this approach is valid for customary evoked potentials during exogenous activation of individual major pathways [11,20,22,23,24,25], it cannot be applied to ongoing LFPs. The CSD of oscillatory LFPs always exhibits a temporal succession of sinks and sources in both the active and passive domains [5,26,27,28,29,30,31,32]. This provides no information as to the polarity of the synaptic currents and thus, several distinct interpretations are feasible (Figure 1A). In most cases, either the sources or sinks will be spurious, making it difficult to determine the location and polarity of the currents, and to interpret their fluctuations.

Neither theoretical nor experimental techniques alone provide an acceptable solution to the problem described above. Thus, we devised a combined approach that collects all the necessary information and determines the polarity and reliable magnitude of synaptic currents. Here, the analysis of animal data is presented side by side with computer simulations that model LFP recordings in an architectonically realistic aggregate of the CA1 region of the hippocampus [33,34,35]. The parallel processing of animal and model LFPs helps us to understand the scaling-up from single cell currents to aggregate LFPs, and the biophysical basis of the procedure.

## Results

### Rationale and strategy

During ongoing oscillations two key issues must be addressed sequentially: (a) mixed activity induced by several afferent populations must be separated to gain accurate spatial limits for each synaptic input; and (b) the baseline reference missing in standard AC-coupled recordings must be estimated to obtain correct polarity of the input. We previously described the successful resolution of problem “a” [8,35] (see steps 2 and 3 in Methods), while the resolution of problem “b” is described below.

**Figure 2:**
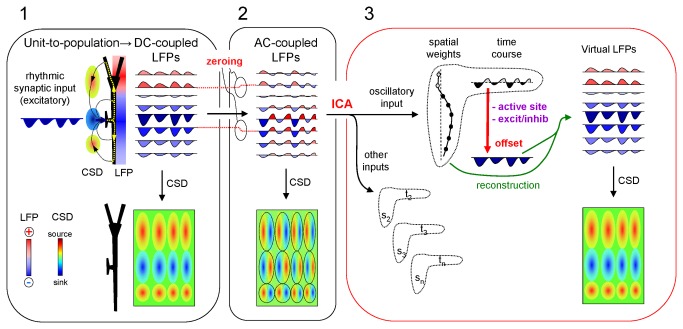
Summary of the procedure and signal transformations. Block 1 illustrates the relationship between the single cell currents and the macroscopic field potentials recorded in ideal DC-coupled mode. A rhythmic excitatory drive into a discrete dendritic domain establishes local sinks of current, surrounded by sources (CSD). These spatially-aligned sources and sinks produce laminar field potentials of uneven strength and polarity, whose CSD analysis renders a correct estimation of sources and sinks amplitudes and locations. Block 2 illustrates the effect of AC-coupling on recordings. Note that channels are individually filtered (*zeroing*) and thus each is offset by a different amount (red dotted lines). Consequently, each gamma wave is transformed into a biphasic sequence at any location. CSD analysis of the AC-coupled profile results in spurious sequences of sources and sinks at all sites. Each LFP wave (dashed ovals) returns a source/sink pair, while the lack of a true reference baseline confounds their initiation time and does not allow us to ascertain which of sources or sinks are expected in cell domains. Block 3 illustrates the rectification procedure. First the ICA decomposes the original signals into pathway-specific generators (pairs s_1_t_1_. s_n_t_n_; only one is used for simplification), each with a spatial distribution and a temporal activation (time course). Note that the curve of spatial distribution is proportional to the collection of offsets introduced by AC-coupling (*zeroing* in Block 2), whilst the time evolution is unique. The experimental determination of the active synaptic sites and the excitatory/inhibitory chemical nature allows offsetting the time envelope as required in order to achieve homogeneous polarity (red vertical arrow). The subsequent reconstruction using the rectified time envelope (green curved arrows) regenerates LFPs with the correct baseline at each recording site. Consequently, the application of CSD analysis generates spatiotemporal maps of sources and sinks in which individual waves have the correct amplitude and duration.

Let us first illustrate the issue by employing numerical simulations to solve the forward problem (Figure 1B), i.e. how to evaluate LFPs from synaptic currents in specific domains of the cellular units (see model details in Methods). It should be noted that both excitatory and inhibitory synaptic activation of discrete cell domains (e.g., thick arrows in Figure 1B1) create loops of inward and outward transmembrane currents that span the entire cell anatomy. In the population, these loops appear macroscopically segregated into different strata corresponding to homologous cell domains, termed *active* for the sites of synaptic currents and *passive* for their surrounding domains. Thus, the resulting LFPs in the aggregate *follow the temporal but not the spatial pattern* of synaptic currents. To illustrate this we simulated an excitatory input (Figure 1B1, *I*_*syn*_) to a dendritic band in the apical tree of a model pyramidal neuron (Figure 1B2, red compartments). A population of these neurons generated oscillatory LFPs that displayed positive or negative polarities in different spatial domains (Figure 1B3). Indeed, LFPs may be of opposing polarity at sites equally spaced from the active zone (compare LFPs labeled by arrows). Thus, direct observation of an LFP cannot be used to infer the location and polarity of the synaptic currents. The problem is further complicated for real LFPs recorded in AC-coupled mode (Figure 1A). Filtering removes or severely distorts useful landmarks provided by slow and DC components, and only fast waves (usually > 0.1 Hz) remain in the recording. Therefore, a given LFP oscillation could have been generated by either pure excitatory (Figure 1A, option 1) or inhibitory (option 3) inputs, or by an alternating succession of excitatory and inhibitory inputs (option 2). The difference between LFPs recorded in AC or DC-coupled modes and the estimation of their respective underlying currents by CSD are illustrated in Figure 2 (blocks 1 and 2).

The above uncertainties burden heavily the cellular interpretation of LFPs. The most outstanding ones are illustrated in Figure 1C and 1D. Standard AC-coupled LFPs were recorded simultaneously along a linear track spanning the CA1 field of the rat hippocampus (e.g. Figure 1A). These exhibited bouts of activity of variable duration but with a characteristic laminar distribution. In the CA1, small amplitude gamma oscillations appear in a rather stable manner in the zone where Schaffer collaterals terminate in the st. *radiatum*. However, when these laminar LFPs are analyzed by CSD to estimate the underlying population synaptic currents (Figure 1C), one can appreciate notable divergence in amplitude and even polarity of individual gamma waves recorded in the Schaffer band (trace superimposed in the CSD map) compared to the currents estimated at the same site. Note that some waves had no associated current sink while in others the net current was reversed to a source (compare the waves labeled with triangles). The disparity between the LFP and CSD at a given site was associated with the presence of non-coherent currents in other strata (e.g., the sink at the st. lacunosum-moleculare marked with an arrow in Figure 1C), the intensity and polarity of which affected the net current of individual waves in the location of Schaffer terminals.

Such discrepancy not only prevents a quantitative evaluation of ongoing LFP waves in terms of compounded output of an afferent population, but also reduces the possibilities of identifying the population by correlation with firing of units. Indeed, the temporal relation of the spike trains from single units to LFPs simultaneously recorded in different strata revealed different phase relations (Figure 1D). In consequence, the position of the unit relative to the LFPs (pre- or postsynaptic) cannot be safely determined.

We propose a series of signal transformations to recover the lost/hidden information (Figure 2). Blocks 1 and 2 illustrate the scaling of single cell currents to macroscopic field potentials and the distortion introduced by recording in AC-coupled mode, respectively (note the different amount of DC potential removed at different sites within the same profile). Our approach consists of five steps:

1. Simultaneous recording of LFP profiles along the main axis of CA1 pyramidal cells (Figure 2, block 2).
2. Separation of pathway-specific contributions (*problem a*) for which we use a combination of previously described mathematical treatments of LFPs including Independent Component Analysis (ICA) [8,35,36] (transition between blocks 2 and 3: see Methods).
3. Estimation of the dendritic domains of transmembrane currents set by the oscillatory input of interest using CSD analysis of its reconstructed (virtual) LFPs.
4. Identification of active (synaptic) domains and their excitatory/inhibitory nature using local microinjection of transmitter blockers at each of the dendritic domains.
5. Rectification of CSD maps by offsetting according to the polarity of the active currents. The steps 3 to 5 are represented by the red vertical arrow in block 3 of Figure 2.

For the purpose of illustration, we selected the ongoing Schaffer input to the CA1, *ignoring a priori knowledge of its excitatory nature*. In anesthetized animals the Schaffer input constitutes a steady sequence of discrete field events within the gamma frequency [6,37]. We also applied the procedure to model LFPs and compared the statistical parameters with the experimental data side by side.

### The CSD of pathway-specific LFPs exhibits stable spatial domains (steps 2 & 3)

**Figure 3:**
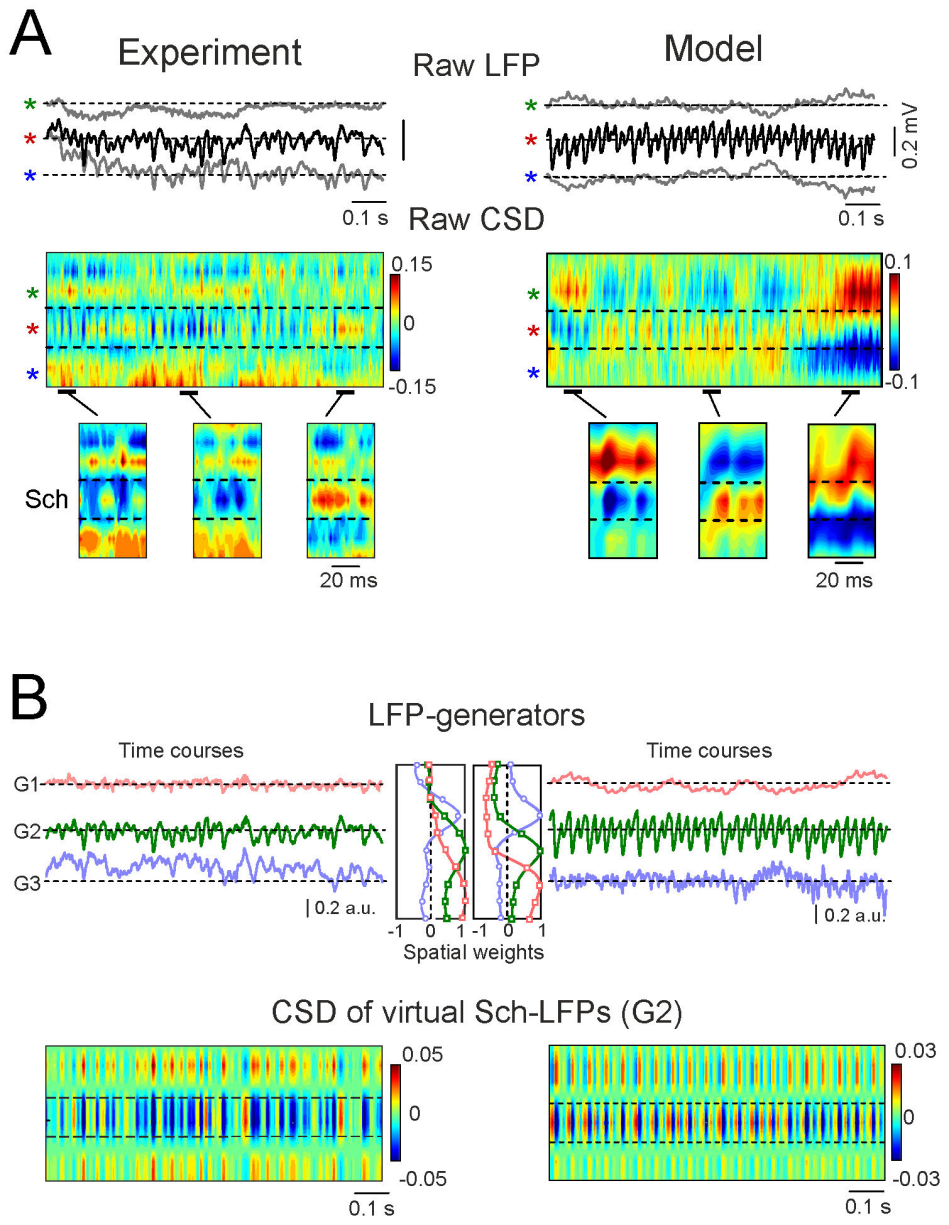
Isolation of currents containing contributions from an oscillatory LFP of interest. Application of the procedure to experimental and model LFPs are presented side by side to facilitate the biophysical interpretation of signal transformations (left and right columns, respectively). A shows sample traces of raw LFPs at different domains along the pyramidal cell axis: colored stars mark recordings in the st. pyr, rad. and l-m, respectively. Model LFPs were high-pass filtered (>0.1 Hz) to reproduce AC-coupling of experimental recordings, and the distribution of the inputs was simpler than in the real CA1 to better visualize the changes in oscillatory input. CSD analysis of LFPs produced a complex spatiotemporal mixture of current sinks and sources. Few or no domains of active synaptic sites (cf. Figure 1) were detected. The amplified segments below show that individual gamma waves in the st. rad. may be matched by either sources or sinks at the active Schaffer domain. B shows the separation of the pathway-specific contributions by ICA applied to raw LFP profiles. Three different generators (G1-G3) were obtained, each of which defined by the characteristic spatial distribution (weight at each electrode) and temporal activation specific to the period analyzed. The respective spatial distributions are shown in the middle. Those obtained for model LFPs tightly reproduced the distribution of synaptically activated compartments, and the temporal sequence of inputs was accurate. Following reconstruction of pathway-specific LFP profiles, the application of CSD analysis rendered a spatiotemporal map of sources and sinks in which stable reversal sites were observed. The model confirmed that these corresponded to the macroscopic boundaries of active and passive domains of the synaptic input. However, note that both sinks and sources still appeared at the synaptic site. These temporal patterns do not allow us to determine whether sinks or sources at each domain are real or spurious.

Although the activation of a single pathway should yield a stratified distribution of sources and sinks in specific cell domains [37], this is not normally evident during ongoing activity contributed by multiple pathways (Figure 3A). The data from the model confirmed that the blurring of expected subcellular domains of current sources and sinks arises due to the overlapping temporal activation of multiple synaptic inputs arriving at different dendritic loci of the pyramidal cells (Figure 3A, right column).

To identify pathway-specific contributions to the LFPs (*Step 2*) we applied the ICA (see Methods) to long epochs of linear recordings. Consistent with our previous findings [7,36], three different components or LFP-generators were obtained in the CA1 region (Figure 3B). We selected the CA3-CA1 Schaffer input (G2) due to its stable development of gamma oscillations of notable amplitude fluctuation in the anesthetized animal [6]. The gamma waves in the Schaffer-generator are produced by postsynaptic currents elicited in the CA1 pyramidal population by the synchronous firing of upstream CA3 pyramidal cell clusters, thus termed micro-field EPSPs (µ-fEPSPs) [6]. For clarity, we simulated in the model a much more regular gamma oscillation and used a sharper spatial distribution of the input (Figure 3B).

We next reconstructed virtual Schaffer-specific LFPs (see Methods), which contained part of the raw LFPs if the corresponding pathway alone was active. In contrast to the raw LFPs, the pathway-specific LFPs displayed full laminar coherence and their amplitudes were proportional at any given time instant. The CSD (*Step 3*) revealed two significant properties (Figure 3B, lower panels): 1) mixed current sinks and sources; and 2) clear stratification over the pyramidal cell axis in both experimental and model data (dashed lines in CSDs). The first property can be explained by the unknown (filtered out) baseline, while the second one was confirmed to reflect natural boundaries of active and passive dendritic domains in the model. The emergence of cellular domains for Schaffer-associated currents indicated that the temporal variation in the spatial shifts introduced by concomitant inputs had been corrected, but we had yet to define active and passive domains, and determine the correct polarity.

### Chemical identification of the active synaptic domain (step 4)

**Figure 4:**
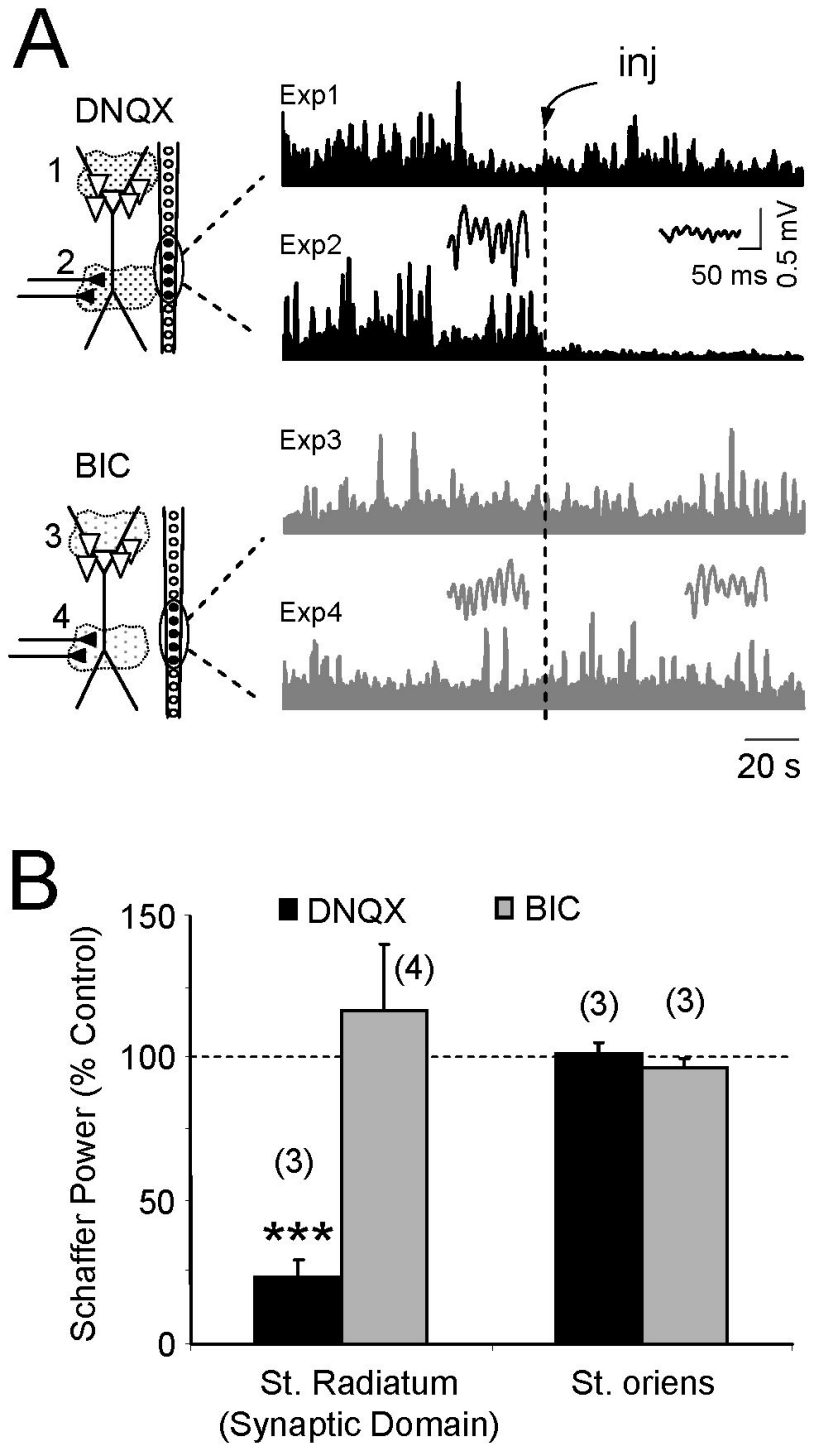
Experimental localization of the active synaptic domain and determination of its chemical nature. Excitatory and inhibitory neurotransmitter blockers were injected locally, one at a time, near the linear probe at the sites located in the spatial domains found after CSD analysis of virtual Schaffer LFPs (A). DNQX and bicuculline (BIC) were injected in the st. pyr./st. or (Experiments 1 and 3). or the st. rad. (Experiments 2 and 4). The plots illustrate the temporal envelopes of the activity of the Schaffer generator obtained following ICA of the LFP profiles before and after drug injection (inj). The insets show sample epochs of virtual Schaffer LFPs. Note that only the Glu-receptor blocker DNQX abolished the activity of this generator when applied in the st. rad. (Expt. 2). All experiments belong to the same animal. B shows the population data (mean ± s.e.m.; *n* is the number of animals): ***p<0.001, Student’s t-test.

The identification of the active domains and the excitatory/inhibitory nature of the synaptic currents were based on the assumption that only one type of blocker should exert an effect when applied at the active domain, i.e., the sites at which postsynaptic receptors are located. Thus, we injected DNQX or bicuculin (BIC) to block either Glutamate or GABA receptors, respectively, at the cell domains identified in step 3. Examples with blockers injected into the st. pyramidale/oriens and into the center of the middle band (st. *radiatum*) are shown in Figure 4A for the same animal. DNQX had no effect when applied to the st. pyramidale/oriens (Figure 4A, Expt. 1) and likewise, BIC did not alter the power of the Schaffer-specific LFPs at any location (Experiments 3 and 4). Only DNQX suppressed the oscillations when applied to the center of the middle apical dendritic band (Expt. 2). Based on these findings we conclude that the middle apical dendritic domain (blindly determined by the CSD of virtual LFPs) is active and receives excitatory input, as supported by the results from other animals (75% reduction for DNQX in st. *radiatum*, n = 3 animals; Student’s *t*-test, p < 0.001; Figure 4B).

### Rectification of synaptic currents (step 5)

**Figure 5:**
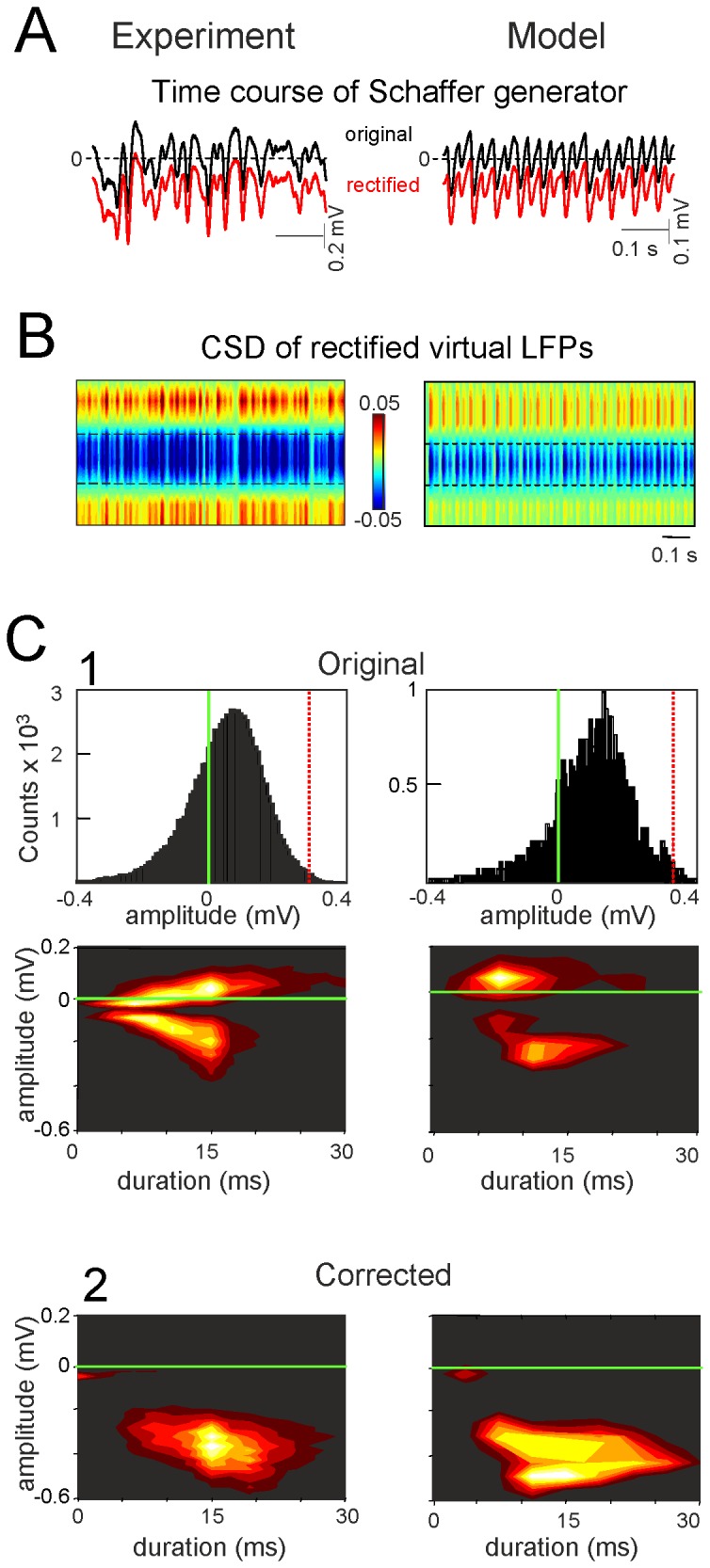
Baseline rectification of the temporal envelope leads to clean CSD bands in virtual LFPs. The temporal activation of the epochs analyzed was rectified by a constant amount and polarity, as determined experimentally (A). This process enabled the lost DC components to be restored at each recording site by subsequently reconstructing virtual LFPs. Since the corrected LFPs are proportional to the underlying current sinks, either one can be used for quantitative estimation of the individual wave parameters. CSD analysis of rectified virtual LFPs in the experiment and the model (B) renders clean bands of sinks at the synaptic domain of the Schaffer input and surrounding sources in the st. pyr and l-m (the epoch corresponds to the same segments as in Figure 3). C shows the estimation of the amplitude and duration of individual gamma waves before (1) and after (2) correction in long experimental (600 s) and model epochs (450 s). The distribution of amplitudes in raw experimental and model LFPs revealed absolute positive and negative values for the individual waves due to the ongoing offset in raw LFPs, which turned their absolute values to biphasic or pure positive values. Following correction, both the absolute amplitude became solely negative and they increased. The densitograms below show the distribution of the amplitude/duration pairs accumulated from individual waves in representative experiments and simulations. Note that after correction, the cloud is not a simple transposition in the two axes, as individual dots (waves) may have required different degrees of correction.

According to the field theory, extracellular currents at a synaptic site exhibit a unique polarity regardless of the temporal activation, while those in surrounding cell domains must be of the opposite polarity (return currents). As we demonstrated that the input was excitatory in step 4, the net CSD for this LFP-generator in the active band must be negative at any time instant, i.e., we should have only sinks (inward currents) in the synaptic domain and not sources (outward currents). Thus, we rectified the time course of the Schaffer LFP-generator (G2 in Figure 3A3 and 3B3) by applying an offset to the temporal envelope in order to maintain its negative polarity (Figure 5A). In other cell domains, the amount and sign of the offset removed by AC filtering of native LFPs may differ since it may have been contributed by different pathways. Importantly, the offsets corresponding to a single pathway in different recording sites must be proportional at any instant and jointly reproduce the same spatial distribution of the AC-coupled pathway-specific Schaffer LFPs (Figure 3B). Therefore, reconstruction of virtual LFPs using the corrected time course and the spatial distribution of the visible (AC-coupled) part of the Schaffer generator introduces the necessary offset in each channel; sinks only appear in the synaptic band and sources in the surrounding domains (compare lower panels in Figure 3B with Figure 5B, before and after rectification, respectively). Moreover, we noticed that the sequential appearance of sinks and sources no longer occurred. Accordingly, negative-going LFP-waves were matched with pure sinks in the synaptic domain and they maintained parallel time-courses, as expected. Although the absolute value of individual LFP-waves is unreliable in raw potentials, the rectified CSD provides a correct estimation of the magnitude of the activation of the corresponding synaptic pathway.

### Determining the true value and polarity of ongoing gamma waves

From the rectified currents in the synaptic domain we were able to quantify the error while estimating the CSD from raw LFPs. When the distribution of the amplitudes of synaptic events (µ-fEPSPs) in the Schaffer band (upper histograms) and the combined amplitude/duration distributions (lower densitograms) were plotted (Figure 5C1), the events had positive (sources) or negative (sinks) polarity as they were measured from baseline. The epoch analyzed yielded distribution that peaked at 0.1 mV in amplitude and 13 ms in duration. After rectification all the waves of the CSD became pure sinks (negative), and both the mean amplitude and duration increased to -0.4 mV and 15 ms, respectively (Figure 5C2). Notably, identical results were obtained in numerical simulations. It should be noted that the entire process corrected individual waves by different amounts, as each had been offset variably according to the concomitant inputs.

### Reliable classification of pre and postsynaptic units by spike-phase correlations

**Figure 6:**
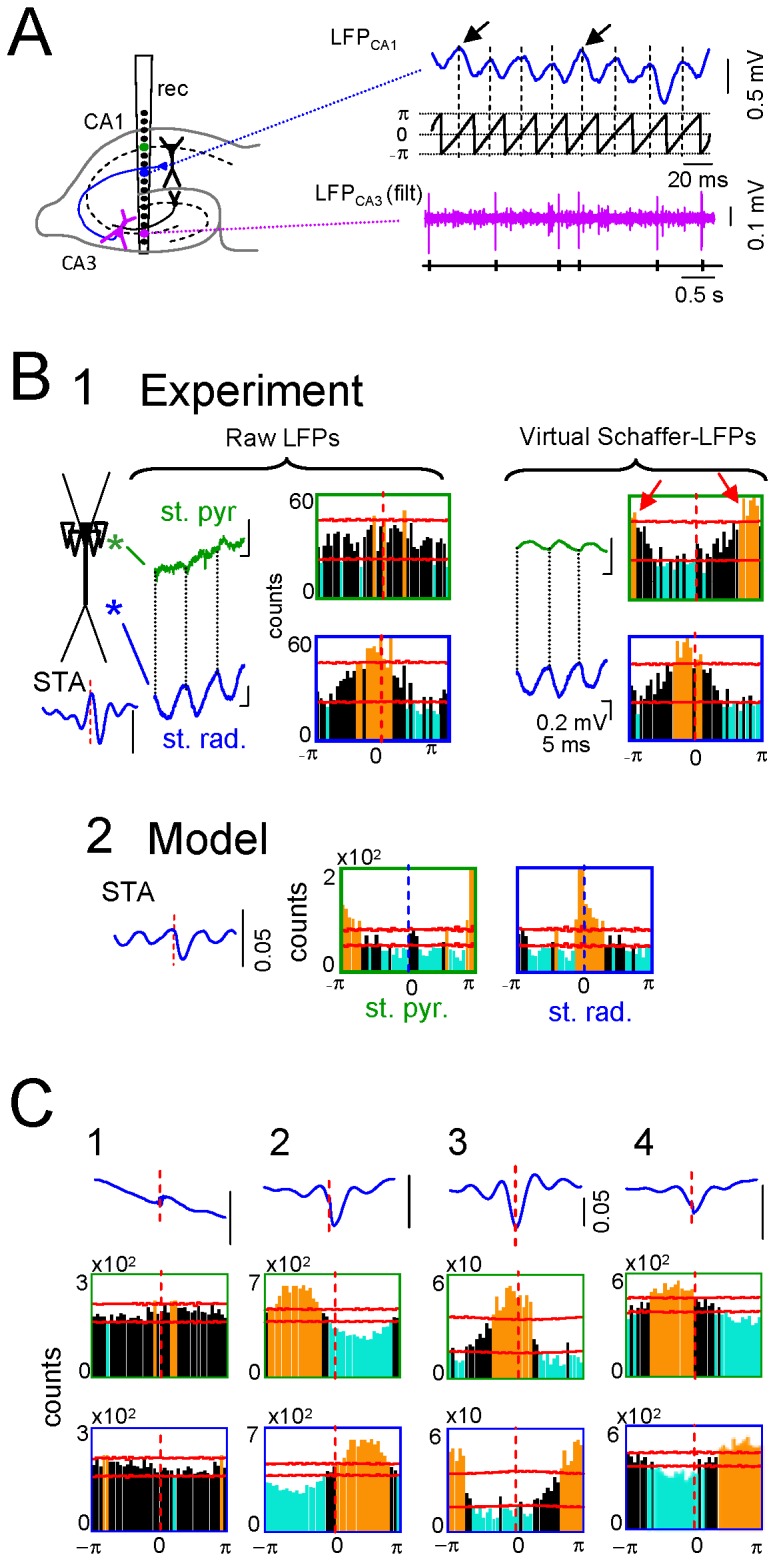
Setting the initiation time of individual gamma waves discriminates the afferent and targeted neurons. A shows the pathway that produced an oscillatory LFP, and the electrode arrangement. A single linear probe spanning the CA1 and CA3 regions can be used to extract and rectify the Schaffer LFPs in the CA1, and to isolate units in the afferent CA3 and postsynaptic CA1 regions. A corrected fragment of the virtual Schaffer LFP in the st. rad. is shown in blue and the phase of the Hilbert transformation in black. The beginning of each wave can only be safely set at the synaptic domain in pathway-specific LFPs of known polarity (arrows), which are then taken as zero. Spike-phase histograms are built against the point processes from spike trains. Electrophysiologically identified CA3 pyramidal cells showed a significant STA of the virtual Schaffer LFPs and characteristic phase locking that was site-dependent (B1): close to zero with respect to virtual LFPs in the synaptic domain (blue traces and boxed histograms) and half-cycle shifted for those in the st. pyr./or. (red arrows). Identical results were obtained in numerical simulations when cross correlating model LFPs and the timings used for synaptic activation (B2). However, no significant phase coupling was found for raw LFPs at the st. pyr./or. (left histograms), which presented undefined phases (compare green and blue traces). C: Most neurons in the CA1, identified as putative interneurons, did not show significant STAs or spike-phase relationships (e.g., cell 1). A fraction of these neurons (37%) recorded at different vertical locations in the postsynaptic CA1 region, still exhibited a significant site-dependent correlation with virtual LFPs, although this was centered in different phases (cells 2-4). Orange and blue bars indicate significant differences (above or below the mean value, respectively; horizontal red lines indicate the level of significance; bin size = 9°). Note the different phases of the STAs in cells 2-4.

The rectification procedure described above enables the zero reference (beginning) to be defined for each gamma wave in the time course of the afferent input. We built spike-phase correlations of the phase of gamma waves (with and without correction) in the st. *radiatum* of the (postsynaptic) CA1 region and the spike trains of pyramidal cells in the ipsilateral afferent CA3 region (Figure 6A), as well as with cells found in the CA1 itself. Globally, the phase-spike correlation significantly depended on whether we used raw (Figure 5B, left) or Schaffer-specific LFPs (right), yet more importantly, we observed a prominent site-dependent character of the phase-spike histograms.

CA3 cells (28 of 29 identified as pyramidal neurons; 15 min epochs; n = 11 animals) showed a significant spike triggered average (STA) for the raw LFP in the postsynaptic Schaffer band (Figure 6B1), indicating a significant association of the LFP in CA1 to spikes generated by these cells [6]. We observed a significant phase-correlation of these cells with raw LFPs in the st. *radiatum* (Figure 6B1, orange bars in blue-boxed histogram, 7.6 ± 3.1°, mean ± s.e.m.). However, this correlation disappeared at sites of return currents that were influenced by other inputs (Figure 6B1, green-boxed histogram, st. pyramidale/oriens). Evaluating the phase-spike histograms over virtual Schaffer-LFPs returned correct correlations. Besides the phase coupling near 0° in the synaptic band (3.9 ± 3.2°; blue-boxed histogram), we also observed significant coupling at 180° (191 ± 3.2°; green-boxed histogram, red arrows) that corresponded to phase-shifted return currents, as predicted by the field theory (Figure 6B1, note phases of green and blue traces). These experimental correlations were supported by the numerical simulations (Figure 6B2). The slight deviation of the phase in simulated relative to the experimental results is due to the absence of natural synaptic delays and the temporal jitter of individual spikes in the model.

No relationship with the Schaffer gamma waves (i.e., postsynaptic to Schaffer-LFPs) was evident for the majority of putative interneurons recorded along the CA1 region, as clearly shown in four representative examples of the phase-spike correlations (e.g. cell 1 in Figure 6C). We found only 9 out of 24 cells with significant STAs and phase coupling to virtual Schaffer LFPs, yet with a relatively large dispersion within the gamma cycle (e.g., cells 2-4). While zero-phase coupling (with slight positive delay due to spike conduction) is expected for afferent CA3 pyramidal cells, the phase dispersion of CA1 units indicates the mono- or polysynaptic drive of these units by the Schaffer input.

## Discussion

The use of LFPs to reflect neural activity requires two main obstacles to be overcome: the AC-coupling of recordings and the temporal variation when blending electrical fields produced by co-activated synaptic inputs. We show that by combining the segregation of pathway-specific contributions to LFPs with local pharmacology we can localize synaptic domains to the cell generators and determine the polarity of synaptic currents. This information allows us to rectify temporal fluctuations in the oscillatory component of the baseline, and to recover the correct polarity and magnitude of membrane currents at any given time point or location. As a result, amplitude fluctuations of successive waves can be interpreted as the natural variations in afferent activity in homogeneous upstream excitatory or inhibitory populations. This approach also allows us to identify the beginning of each wave, which facilitates their matching to spike trains. Accordingly, the afferent cells of origin exhibit a significant phase relationship at fixed positive values while postsynaptic units activated by the input show varying phases according to mono or polysynaptic delays.

An essential step in the procedure is to determine the site and chemical nature of the active synapses in experiments, such as in the excitatory Schaffer pathway studied here. Although the procedure can be generalized to other pathways and LFPs, each may require ad hoc modifications and/or further testing in complementary experiments. For instance, one may anticipate some complications for inhibitory-based oscillatory LFPs. Thus, the use of GABA blockers typically initiates epileptic activity that may profoundly alter network activity and hence, the synaptic composition of LFPs [7]. Nevertheless, this step is formally qualitative, that is the procedure does not require the analysis of LFPs after transmitter blockade but it is only used to find out whether it is effective in reducing the oscillatory LFP of interest in a specific subcellular domain, given that the offset for baseline correction is determined empirically on the crude temporal envelope of the ICA-isolated oscillatory component. It may happen that a given oscillatory LFP is blocked by both Glu and GABA antagonism in the same or different subcellular domains, such as would occur by the blockade of an excitatory drive to a population of interneurons ultimately producing the inhibitory LFP oscillation [32]. In such cases it is crucial to ensure that only one pathway is contributing to the currents underlying the LFPs and thus, mathematical separation becomes more critical. Normally, the excitatory drive to multipolar interneurons does not produce any significant LFP. Anatomical data precisely describing the different territories of interneuron dendrites, and of their axonal contacts onto principal cells, may help guide pharmacological intervention. There may also be cases in which two afferent pathways overlap so tightly on the same subcellular domain of target populations that their separation by ICA methods is unfeasible (e.g., the mixture currents in the CA1 st. lacunosum-moleculare [7]). In this situation, the present approach will not be efficient as it requires full pathway-specificity. Still, it may help to know if only one or both afferent populations send an oscillatory patterned output, as the present approach could be further complemented with additional techniques for separation, such as temporal ICA or Fourier-based methods.

Realistic modeling of LFPs helps to understand the scaling of single-cell currents to macroscopic fields in volumes within a common biophysical framework [33,34]. Here we use these realistic models to allow compartmental currents interact freely in function of the channel kinetics and the morphoelectronic structure of the cellular units, thereby showing that the mathematical treatment of compound signals is not a statistical construct but rather, that it truly derives from the electrochemical nature of the signals, and their interplay in cell and volume conductors. We previously verified that within plausible physiological ranges of unitary settings, LFP reproduction from subthreshold synaptic inputs is consistent [35]. However, substantial differences can be expected through sustained regional changes in membrane conductance, such as those produced by synaptic bombardment in afferent pathways missing from the model. However, for the present purposes the most relevant setting is the architecture of the population, which makes it only applicable to LFPs in the CA1 region. A truthful cytoarchitecture is essential, since cell geometry and the population curvatures can severely distort LFPs [14,17,19] to the extent that they may reach a larger amplitude even far from the generating cell elements, such as in the hilus of the Dentate Gyrus [38]. In curved structures, a careful CSD is crucial to finely discriminate active from passive somatodendritic domains.

The variety of spatiotemporal combinations brought about by the activation of different pathways that contribute to field oscillations and LFPs in general is not amenable to approaches based on frequency decomposition alone [12,39], given that frequency domain methods cannot detect the shifting activation/deactivation of multiple current generators. Indeed, the variability and inconsistency of the frequency bands recorded in the same or different cortical area(s) suggest that this problem may be more widespread than generally suspected [40,41]. It is also common to build depth profiles of the waves averaged against an arbitrarily chosen phase [42]. However, this approach does not capture the dynamic information contained in wave fluctuations. Other procedures seek the partialization of recordings relative to a distant point (partial coherence analysis) [43,44], which allows a degree of spatial and temporal segregation of activity [9], but does not solve the problems related to polarity of the underlying synaptic currents. One might also think that estimating the CSD of ongoing LFPs would be sufficient to discriminate both the polarity and the contribution of multiple pathways. Indeed, CSD analysis does remove volume-conducted currents, although it cannot separate the mixed currents elicited by multiple generators within the recording zone. Active and passive sinks and sources elicited by pathways converging on pyramidal cells exert an envelope effect on one other [6,7] to the extent that they become unrecognizable in some epochs, thus polarity is unreliable. Although several approaches have been proposed to identify stable spatial domains (e.g., applying ICA to spatiotemporal maps of CSD), these methods are not very efficient when dealing with physiological data [45].

AC-coupling of standard amplifiers introduces spatial bias by subtracting the mean value from each recording (cf. in Figure 2). ICA of LFPs efficiently cleans concomitant inputs and renders pathway-specific components in which the AC-coupling-related bias remains. We have shown that the AC-bias can be easily corrected for each LFP-generator separately. The key step is the spatial and temporal dissociation of LFP generators extracted by ICA. Thus, by introducing an offset on the temporal component only, the subsequent reconstruction enters a weighed offset in each recording site such that cell domains achieve homogeneous current polarity. Although the DC component cannot be restored precisely, the rectified currents fulfill the criteria for the spatial distribution of sub-threshold synaptic currents elicited by neuronal generators: (1) the instantaneous balance of sources and sinks (net generator current equals zero); (2) a unique macroscopic polarity of currents specific for each of the active and passive domains over time. Therefore, the currents underlying each wave gain reliable proportional amplitude over time. The most plausible interpretation of LFPs relates the amplitude of any wave with the number and degree of synchronization of the afferent units that fired together in the upstream population [7,16]. We previously showed that each gamma wave in the Schaffer LFP-generator represents excitatory input from a different afferent cluster of CA3 pyramidal cells, and that the CA1 pyramidal cells fired more frequently when time-locked to the larger waves [6]. Accordingly, wave fluctuations appear to reflect the size of the successive cell clusters.

Our procedure to rectify the time envelopes of LFP-generators enables the initiation of each wave to be defined with reasonable precision. This cannot be performed for raw LFPs, for which arbitrary phases are routinely employed. Our approach permits the dynamic interactions between afferent and postsynaptic populations to be explored [6,7,37], allowing phase-spike correlations to then be used to discriminate afferent from driven cells. Afferent units maintain a near zero phase with the postsynaptic currents (as in the model or the corrected CSD at the synaptic domain), whereas cells driven by them should exhibit shifted phase coupling according to the EPSP-to-spike lag.

The study of LFP oscillations is largely focused in understanding the cellular and network mechanisms setting and/or contributing to the global pace or to the phase of individual waves in relation to neuron firing [46], while less attention has been paid to the temporal coding inherent to wave fluctuations themselves. The present approach opens the possibility of correcting for some technical drawbacks that have made unreliable such enterprise. Although it is optimized for LFPs obtained in cytoarchitectonically ordered aggregates such as the hippocampal CA1, the essentially blind nature of all steps makes it applicable to other LFP oscillations and structures. Even if the extraction of the pathway-specific components may require ad hoc treatment of signals, the corrected time course is free of spatial constraints and the internal fluctuations can be safely associated to instant variations in the level of activity of upstream neurons of origin. In future work, this may be tested by exploring in excitatory LFP oscillations the efficiency of waves of different amplitude to fire target neurons. Another interesting perspective is the use of corrected pathway-specific LFP waves in two or more converging inputs to explore how the output code in single cells is elaborated from discrete parallel inputs to dendritic sites not normally accessible to intracellular recordings, and it can be made even in awaken animals.

## Materials and Methods

### 1. Ethics

All experiments were performed in accordance with European Union guidelines (86/609/EU) and Spanish regulations (BOE 67/8509-12, 1988) regarding the use of laboratory animals, and the experimental protocols were approved by the Research Committee of the Cajal Institute (Permit BFU2010-19192).

### 2. Experimental procedures

Adult female Sprague-Dawley rats were anesthetized with urethane (1.2 g/kg i.p.) and placed in a stereotaxic device. Surgical and stereotaxic procedures were performed as described previously [47,48]. Concentric stimulating electrodes were placed in the CA3 region ipsilateral to recording to activate Schaffer collaterals in the CA1 field. Multisite silicon probes (Neuronexus, Ann Arbor, MI) of 16 linear recording sites were used to record at 50 µm steps parallel to the main axis of the CA1 pyramidal cells (AP, 4.5-6.5; L, 2.6-3.5 mm). Linear probes were soaked in 1,1′-dioctadecyl-3,3,3',3'-tetramethylindocarbocyanine perchlorate (DiI) before insertion (Molecular Probes, Invitrogen, Carlsbad, CA) to assess their placement in histological sections post-mortem. A silver chloride wire implanted in the skin of the neck served as a reference for recordings. Signals were amplified and acquired using MultiChannel System (Reutlingen, Germany), and Axon (Molecular Devices, Sunnyvale, CA) hardware and software (20-50 kHz sampling rate).

The excitatory/inhibitory chemical nature of LFP generators was studied by local application of neurotransmitter blockers via glass recording pipettes (7-10 µm at the tip). These were introduced at a 10° angle from the vertical axis and targeted loci within 300-500 µm of the linear probe at different strata of the CA1. Microdrops (50-100 pl) were adjusted to limit drug effects to within 500 µm, as determined by the selective modulation of evoked potentials in the desired group of recording sites. Bicuculline methiodide (BIC) (Sigma, St. Louis, MO) or 6,7-dinitro-quinoline-2,3-dione (DNQX; Tocris, Bristol, UK) were loaded into the pipettes to block GABA-A and non-NMDA Glu receptors, respectively. Drugs were dissolved in ACSF at concentrations ~50 times higher than those usually employed *in vitro*. A single injection ensured stable effects of the drug for at least 60 s.

At the end of each experiment the animals were perfused through the abdominal aorta with PBS containing heparin (0.1%) followed by paraformaldehyde (4%), and the animal’s brains were processed for microscopic inspection (sections were stained with either toluidine blue or cresyl violet and examined by fluorescence microscopy).

### 3. Isolation of ongoing activity from a single pathway (steps 2 and 3)

The ICA provides spatially stable components of coherent activity. While the ascription of ICA components to their source populations and pathways is a challenging problem when recording from a distance, the in-source recording of intra-hippocampal LFPs allows the thorough spatial inspection of active neurons down to the subcellular definition, and direct matching to anatomy as well as to customary spatial profiles of evoked potentials [36].

#### 3.1 Independent component and current source density analyses of LFPs

Detailed procedures have been described previously [6,8]. Mathematical validation and interpretation of ICA components in laminated structures were also performed using realistic LFP modeling [35]. Briefly, *M* simultaneously recorded LFP signals are represented as the weighted sum of the activities of *N* neuronal sources or LFP generators:

$$u_{m}\left(t\right)=\sum_{n=1}^{N}V_{mn}\;\;s_{n}\left(t\right),\;m=1,2,...,M$$ (1)

where {*V_mn_*} is the mixing matrix composed of the so-called voltage loadings or spatial distributions of all LFP-generators and *s*_*n*_(*t*) is the time course of the *n-th* LFP-generator. As the location of recording sites is known, the joint curve of spatial weights of an LFP-generator equals to instant depth profiles of proportional voltage amongst sites, as during laminar recording of standard pathway-specific evoked potentials. To perform the ICA we employed the KDICA algorithm [49], which returns the activations {*s_n_(t)*} and spatial weights {*V_mn_*} of up to *M* LFP-generators. Once extracted, each LFP-generator can be analyzed independently by re-constructing its virtual LFPs, *u*_*j*_(*t*)= *V*_*j*_ *s*_*j*_(*t*). An important preprocessing step is the use of the principal component analysis (PCA), which allows reducing the presence of highly variable remote generators [35] and stabilizes the convergence of the ICA to true stable LFP-generators [8,44]. In this study we used automatic PCA reduction maintaining 99.0% of the initial LFP variance.

The CSD analysis [20,21] determines the magnitude and location of the net transmembrane current generated by neuronal elements within a small region of tissue. We used a one-dimensional approach, which calculates the CSD from the voltage gradients along the cell’s axis [23]. Conveniently, the spatial distortion introduced by unbalanced tangential currents is effectively cancelled out by time averaging of the myriads of microscopic currents as if they all were synchronously activated [35]. The curve of spatial weights for each LFP generator is thus, accurate to the subcellular level. Here we assumed that the extracellular space is homogeneous, as the heterogeneity of tissue resistivity at the level of the stratum (st.) pyramidale [50] introduces negligible distortion to the depth profiles when active currents are located in distant dendritic loci. Thus, we assumed a homogeneous resistivity and used arbitrary units for the CSD. For *in source* recordings, the ICA acts as a rejection device for volume-conducted currents [8], although these may appear in other ICA components. Consequently, the second-spatial derivative of the curve of spatial weights of LFP components extracted for ongoing activity matches that of the CSD, which is verified for excitatory pathways using the corresponding evoked potentials [36].

#### 3.2 Identification of synaptic events

The baseline activity of the Schaffer generator is composed of rhythmic excitatory packages of synaptic events or micro-field excitatory postsynaptic potentials (µ-fEPSPs) in the gamma frequency. To retrieve these events we used the Wavelet Transform of the time course of the Schaffer generator *s*(*t*) [6,51]:

$$W\left(a,b\right)=\frac{1}{\sqrt{a}}\int s\left(t\right)\;\Psi \left(\frac{t-b}{a}\right)\;dt$$ (2)

Where Ψ is the Haar mother wavelet (well suited to the detection of short pulses in a signal), *a* is the time scale and *b* is the localization in time. We then rectified the wavelet coefficients using the following equation:

C(a,b)=max(−W(a,b), 0) (3)

The 2D surface obtained describes the local linear fit of the Schaffer-specific LFP by the pulse-like function (Haar) at scale *a* and localization *b*. Large absolute values of *C*(*a*, b) at a given time instant and scale correspond to abrupt pulse-like transitions in *s*(*t*). We can therefore associate these points in the (*b*, a)-plane with singular LFP events. Consequently, the local maxima

$${\left(a,b\right)}_{k}=\overset{\arg max}{\underset{w_{k}}{}}\;\left(C\left(a,b\right)\right)$$ (4)

define the time instants of µ-fEPSPs (given by *t*_*k*_ = *b*_*k*_ -*ak*/2), their duration (given by *a*_*k*_) and their amplitude (given by *A*_*k*_ = *C(a*_*k*_,*b*_*k*_)).

### 4. Spike sorting, unit classification and statistical tests

Spike trains of individual units were obtained from unfiltered recordings of the CA1 and CA3 regions using wavelet-enhanced spike sorting [52] and local CSD methods. As described previously [6,7,37], units were classified into two subclasses, pyramidal cells and putative interneurons, based on the location of the recording site and additional standard electrophysiological criteria [53].

Spike-triggered averages (STAs) of CA1 LFPs were obtained from a spike series of single CA3 units and for putative interneurons in the CA1 region. Trains contained at least 1,500 spikes and the level of significance was determined using the surrogate test (1,000 trains with randomly shuffled inter-event intervals: α ≤ 0.05). The standard Student’s *t*-test was used to analyze the differences between two sample means.

To estimate the correlation of unit firings with the phase of LFP-generators, raw LFPs and reconstructed pathway-specific LFPs, we used the phase provided by the Hilbert transform and constructed a histogram of the phase values corresponding to spike occurrences. The Rayleigh test (*p* < 0.05) for non-uniformity of circular data was used to examine the significance of the unit-source couplings.

### 5. Simulation of ongoing LFPs

Realistic LFPs were simulated as described previously [33,34,35,54]. Briefly, we built a mathematical framework containing the following four coupled components: the spiking activity of afferent populations; the detailed dynamics of single target cells; the architecture of the neuronal aggregate; and the spreading of electrical currents in the extracellular space (Figure 1B).

#### 5.1 Simulation of synaptic inputs

For the sake of completeness we simulated LFPs composed of contributions from multiple synaptic sources in different cell domains in order to perform identical analyses of real and model LFPs, including the extraction of a rhythmic synaptic input from a mixture of complex source composition. In a previous study we demonstrated that inputs contributing a weak variance to the LFP (weak LFP-generators) may suffer from cross-contamination by stronger inputs due to non-linear intracellular interactions of coincident conductances [35]. Thus, we designed a combination of inputs in which our pathway of interest generated a relative variance over the entire set of recordings similar to that found in the experimental results (3-10%) [6,36].

The model LFPs shown here were obtained using a combination of three synaptic inputs in discrete dendritic bands mimicking the stratified input from populations afferent to principal pyramidal cells of the CA1 (colored compartments in the schematic neuron: Figure 1B2): the excitatory Schaffer input from the ipsilateral CA3 region (in red); and two inhibitory inputs, one perisomatic (in green) and another making contact in the distal third of apical dendrites (in blue). These three inputs resemble the anatomical descriptions of the more conspicuous LFP-generators of the CA1 region identified in previous experiments [7,36]. For simplicity, the Schaffer-like input was restricted to a single band in the apical tree. For excitatory and inhibitory inputs, we used the kinetics of non-N-methyl-D-aspartate glutamate and GABA_A_ receptors, respectively.

All target neurons in the aggregate received the same inputs, which ensured that the contributions to the LFPs calculated over a vertical tract at the center of the slab were proportional (Figure 1B3). For each input we simulated ongoing bombardment through afferent axons with either rhythmic (Schaffer input: see sample in Figure 1B1) or random-like (Poisson distribution) spike trains (inhibitory inputs). The perisomatic inhibitory input was homogenously distributed over a dendritic band from 50 µm into the basal tree through to 100 µm into the apical tree (neuronal length = 750 µm), and at a total mean frequency of 300 inputs per second. The apical distal inhibition was applied to cell compartments 400 to 500 µm from the cell soma (equivalent to the st. lacunosum-moleculare, l-m), and the temporal activation was built by blending two independent temporal series of random inputs (joint mean frequency of 260 inputs/sec), in turn modulated with random on/off periods. This procedure permits stronger time fluctuations and hence, a large relative variance is introduced by this input into composite LFPs (>50%) [7]. The Schaffer input was delivered to apical compartments at regular intervals between 150 to 300 µm from the cell soma (40 Hz).

#### 5.2 Single cell model

We simulated the dynamics of a realistic neuron model with the average branching, total dendritic length, and dendritic tapering of CA1 pyramidal neurons, as well as the appropriate variations in spine density described in detailed morphometric studies [55,56,57]. This model neuron has been tested thoroughly elsewhere [33,34,35,54,58,59].

The compartmental model neuron has been described elsewhere in detailed and its cell morphology can be found at http://www.cajal.csic.es/departamentos/herreras-espinosa/ca12011/index.html. The length of the compartments λ was always between >0.01 and <0.2. The total effective area of the neuron was 66,800 µm^2^ (including the spine area), the membrane capacitance (C_m_) was 1 µF/cm^2^, and the internal resistivity (R_i_) was 100 Ω·cm. The membrane resistivity (R_m_) was 50 kΩ·cm^2^ for the soma and it varied in the dendrites (see above URL for details). The input resistance measured at the soma was 60 MΩ and the time constant was 18 ms. Dendritic spines were collapsed into the parent dendrites. As a result, the values of R_m_ and C_m_ of the parent compartments were compensated accordingly. For apical dendrites the surface ratio between the spines and parental dendrites was set to 1:1 [56] and thus, we used a correction factor of two for spiny compartments (i.e., the R_m_ was halved and the C_m_ doubled).

We used twelve different types of ion channels to simulate the active properties of the cell membrane: two transient sodium currents in the axon and soma/dendrites; two calcium currents (high- and low-threshold); one hyperpolarization-activated “h” current; and seven potassium currents. These potassium conductances represented delayed rectifiers (one axonal and one somato/dendritic), a small persistent muscarinic type current, a transient A-type current (one proximal and one distal), a short-duration [Ca]- and a voltage-dependent and long duration [Ca]-dependent current. The conductance variables were described using a Hodgkin-Huxley type formalism (see details of the kinetics in Refs. 58,59 and the URL above). The reversal potentials for ion channels were set to E_Na_ = 50 mV and E_K_ = -90 mV. E_Ca_ was considered variable and dependent on the calcium concentration.

The channel distribution along the cell was tuned to accurately reproduce the unitary and population electrogenesis of the CA1 region [33,59]. In this study the densities of axonal conductances were diminished by a factor of 100 to avoid somato/axonal spike firing, while dendritic recruitment of V-dependent channels was permitted. Such tuning was required to limit cell firing which hampers the interpretation of the intracellular interactions between subthreshold currents.

The synaptic currents were modeled using the following equations:

$$I_{syn}\left(t\right)=g_{syn}\left(t\right)\;\left(V_{m}-E_{syn}\right)$$ (5)

$$g_{syn}\left(t\right)=\overset{}{{\overset{\wedge }{g}}_{syn}\;\left(\frac{t}{\tau_{syn}}\right)}exp\left(1-\frac{t}{\tau_{syn}}\right),\;t>0$$ (6)

with *τ*_*syn*_ values of 2 and 7 ms, and a reversal potential *E*_*syn*_ of 0 and -75 mV for glutamatergic and GABA_A_ inputs, respectively. For the sake of simplicity, the synaptic conductances were homogenously distributed along the surface of all the dendritic branches within the activated band. Conductances of 15 and 35 nS were assigned the perisomatic and distal GABA inputs, respectively. The rhythmic Schaffer input was varied from 2-8 nS to reproduce the varying amplitude of successive excitatory wavelets or µ-fEPSPs in vivo. This range of conductance was normally subthreshold for local dendritic spikes [58].

Neuronal dynamics and transmembrane currents were calculated using the GENESIS simulator [60]. An exponential (explicit) Euler method was used with the integration step of 1 µs.

#### 5.3 Aggregate model

The dorsal CA1 region was modeled as a slab of tissue containing an aggregate of 16,966 morphologically identical units forming a palisade-like planar structure (1 x 1 mm: Figure 1B3). We preserved an experimentally observed cell density of 64 neurons in a 50 x 50 µm antero-lateral lattice [61], with their main axes in parallel and their soma contained in a cell body layer 50 µm thick, arranged as 4 uneven layers with 66% on the apical side, and 22% and 11% in the two basal layers. The dorso-ventral extension was set at 0.8 mm. As we employed homogeneous activation throughout the population of target neurons, the estimation of compartmental currents was made based on a single unit and the activation of the entire population was then mimicked by replicating the currents in all neurons of the aggregate (see below). In selected runs we checked for the possible effects of anatomical cell-to-cell variability by introducing free axial rotation in units, moderate vertical jitter (one layer vs. 4-layered somata distributions), and random vertical jitter (within ± 50 µm) in the coordinates of cell compartments. We had previously checked that the macroscopic averaging dampened microscopic differences [33,34].

#### 5.4 Calculation of model LFPs

We assumed a homogeneous unbounded conductive medium with a constant extracellular conductivity, σ = 0.3 S/m [50]. Thus, the current spread in the extracellular space Ω can be modeled by the Poisson equation:

$$-\sigma \;\Delta \phi \left(x,t\right)=\sum I_{j}\left(t\right)\;\delta \left(x-x_{j}\right),\;x\in \Omega$$ (7)

where *I*_*j*_(*t*)*δ*(*x-x_j_*) are the point transmembrane current sources with amplitude *I*_*j*_(*t*) obtained in the simulation of the dynamics of pyramidal neurons, and the sum extends over all the cells in the aggregate and their compartments. Using the fundamental solution of the Laplace operator in R^3^ we can approximate the potential near to the center of the neuronal slab as:

$$\phi \left(x,t\right)=-\;\;\frac{1}{4\pi \sigma }\sum \frac{I_{j}\left(t\right)}{r_{j}}$$ (8)

where *r*_*j*_ *=║x-x*_*j*_ *║r*_*j*_ is the Euclidian distance to the corresponding compartment. To simulate electrophysiological recordings, we placed 16 virtual recording points h=50 µm apart spanning from 250 µm above to 500 µm below the cell body at the center of the population, in parallel to the somato-dendritic axis. The simulated LFPs are given by

$$u_{k}\left(t\right)=\phi \left(0,0,kh,t\right)$$ (9)

Calculations of LFPs were programmed in custom MatLab code.
